# *Toxoplasma* LIPIN is essential in channeling host lipid fluxes through membrane biogenesis and lipid storage

**DOI:** 10.1038/s41467-021-22956-w

**Published:** 2021-05-17

**Authors:** Sheena Dass, Serena Shunmugam, Laurence Berry, Christophe-Sebastien Arnold, Nicholas J. Katris, Samuel Duley, Fabien Pierrel, Marie-France Cesbron-Delauw, Yoshiki Yamaryo-Botté, Cyrille Y. Botté

**Affiliations:** 1 Apicolipid Team, Institute for Advanced Biosciences, CNRS UMR5309, Université Grenoble Alpes, INSERM U1209, Grenoble, France; 2 Laboratory of Pathogen Host Interactions, UMR 5235, Université de Montpellier, Montpellier, France; 3 Université Grenoble Alpes, CNRS, Grenoble INP, TIMC-IMAG, Grenoble, France

**Keywords:** Lipidomics, Parasite biology, Parasite host response

## Abstract

Apicomplexa are obligate intracellular parasites responsible for major human diseases. Their intracellular survival relies on intense lipid synthesis, which fuels membrane biogenesis. Parasite lipids are generated as an essential combination of fatty acids scavenged from the host and de novo synthesized within the parasite apicoplast. The molecular and metabolic mechanisms allowing regulation and channeling of these fatty acid fluxes for intracellular parasite survival are currently unknown. Here, we identify an essential phosphatidic acid phosphatase in *Toxoplasma gondii*, *Tg*LIPIN, as the central metabolic nexus responsible for controlled lipid synthesis sustaining parasite development. Lipidomics reveal that *Tg*LIPIN controls the synthesis of diacylglycerol and levels of phosphatidic acid that regulates the fine balance of lipids between storage and membrane biogenesis. Using fluxomic approaches, we uncover the first parasite host-scavenged lipidome and show that *Tg*LIPIN prevents parasite death by ‘lipotoxicity’ through effective channeling of host-scavenged fatty acids to storage triacylglycerols and membrane phospholipids.

## Abstract (web summary)

Apicomplexa generate essential lipids as combination of host fatty acids and de novo synthesized within the apicoplast. Here, the authors identify a phosphatidic acid phosphatase in *Toxoplasma gondii*, TgLIPIN, as central for controlled lipid synthesis and define the host-scavenged lipidome.

## Introduction

Apicomplexa includes several pathogenic protists that are responsible for major chronic and infectious diseases with a massive social and economic burden. *Toxoplasma gondii* and *Plasmodium falciparum* parasites are responsible for toxoplasmosis and malaria, respectively. These obligate intracellular parasites have an enormous demand for lipids in maintaining their survival within their human host cells. The utilization of fatty cids (FA) to synthesize complex lipids is an essential determinant for successful host adaptation by these parasites. Apicomplexa meet their high need for lipids through de novo synthesis via type II fatty acid synthesis (FASII) within the apicoplast and via copious salvage directly from the host and extracellular environment^1–6^. Recent data suggest that the tight regulation of FA flux between host, parasite, and its metabolic organelles is particularly vital for intracellular development of these apicomplexa pathogens^4,7^. Metabolic adaptation of the parasite toward host nutritional environment is also important for parasite propagation^4^. For instance, in low nutrient environments, the parasite is able to form giant multivesicular bodies (gMVBs) in the host, which somehow facilitates the parasite’s ability to scavenge lipids in suboptimal nutritional conditions^4^. However, the molecular and metabolic pathways controlling the lipid flux toward parasite membrane biogenesis and storage remain largely unknown.

Phosphatidic acid (PA), the simplest glycerophospholipid, contributes to the regulation of FA flux in apicomplexa, as it is the key intermediate balancing the biosynthesis of both glycerophospholipids and triacylglycerols (TAGs) toward membrane biogenesis and lipid storage in eukaryotes^8^. PA has pleiotropic roles within the parasite: (i) as a signal transducer modulating parasite invasion, motility, and egress, produced by the action of diacylglycerol kinases (DGK1 and DGK2)^9–12^, (ii) as a regulator of lysoPA (LPA)/PA levels for modulating membrane curvature for cytokinesis and endocytosis, formed by acyltransferase ATS2^4^, and (iii) as the central precursor for bulk phospholipid synthesis and membrane biogenesis, made de novo from FA and a glycerol-3-phosphate backbone by the sequential acylation catalyzed by acyltransferases, in the apicoplast/ER pathways formed by ATS1/GPAT and ATS2/AGPAT^4,13^. Further, PA can be catabolized to a key lipid class, diacylglycerol (DAG) by phosphatidic acid phosphatases (PAP), which play vital metabolic functions in eukaryotes^14^. Together, PA and DAG are central lipid precursors from which most lipid classes can be generated de novo. This includes all phospholipids made from PA and DAG via the CDP-DAG from PA and the Kennedy pathway from DAG, respectively^15,16^. DAG is also the precursor for the synthesis of the major storage lipids, TAG.

Since PA is pivotal for parasite lipid metabolism, the enzymes involved in PA metabolism are critical for understanding parasite pathogenesis. One such critical enzyme class is PAP, as it allows to fine-tune the balance between PA and DAG. Apicomplexa parasites possess three putative PAPs, none of which have been characterized to date.

Here, we determined that *Toxoplasma gondii* LIPIN; *Tg*LIPIN is a PAP that localizes to the cytosolic-ER interface and is essential for the intracellular survival of tachyzoites. Its inducible disruption quickly leads to aberrant membrane anomalies, mostly at the IMC and the endomembrane system, causing division defects and parasite death. Lipidomics of the mutant reveals a time-dependent accumulation of PA and free FA (FFA), concomitant with the reduction of DAG, lipid droplets, and storage, TAG. Both lipidomic and cellular membrane phenotypes are aggravated in a high host nutrient environment. The mutant dies from a lipotoxic accumulation of FFA and major membrane phospholipids, specifically PA. Further, we conducted novel fluxomic experiments using U-^13^C glucose-labeled host cells to monitor the host FA scavenging capacity of the parasite, providing the first host-scavenged lipidome of *Toxoplasma*. This approach showed that *Tg*LIPIN channels host FA to maintain an appropriate PA/DAG synthesis ratio*. Tg*LIPIN acts as a metabolic checkpoint, tightly regulating membrane biogenesis versus lipid storage, which is essential for proper intracellular growth division and survival.

## Results

### *Toxoplasma gondii* genome encodes a single lipin, *Tg*LIPIN, which has functional phosphatidate phosphatase activity

**Fig. 1:**
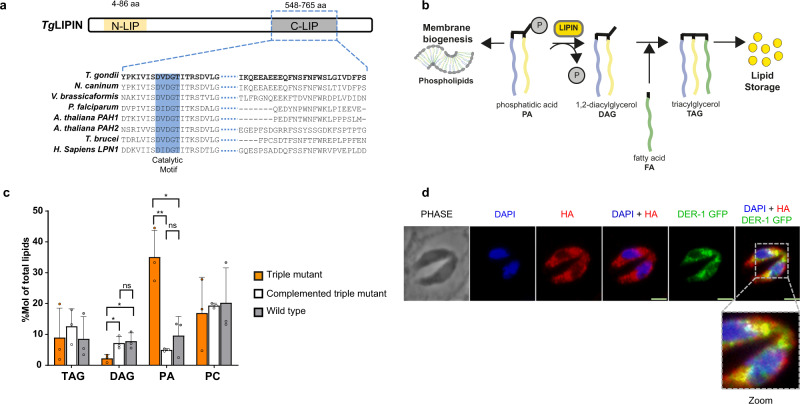
*T. gondii* LIPIN (*Tg*LIPIN) is a phosphatidate phosphatase localized to parasite cytoplasm and endoplasmic reticulum. **a** The C-LIP domain of *Tg*LIPIN is evolutionarily conserved among eukaryotic orthologs and harbors the catalytic motif DVDGT known to be central to PA phosphatase activity. **b** Graphical scheme of the biochemical function of LIPIN in the glycerolipid biosynthesis pathway in eukaryotes. **c** The complementation of the yeast triple KO mutant (*∆dpp1∆lpp1∆pah1*), lacking phosphatidic acid phosphatase activity, with the C-LIP domain of *Tg*LIPIN restores of PA and DAG (*n* = 3, unpaired *t* test *P* values where *P = 0.024 DAG triple mutant vs DAG complemented triple mutant, *P = 0.032 DAG triple mutant vs DAG wild-type, **P = 0.0039 PA triple mutant vs PA complemented triple mutant, *P = 0.039 PA triple mutant vs PA wild type). Data are presented as mean values + /− SEM. **d** IFA of *Tg*LIPIN-HA (endogenous C-terminal tag) with anti-HA, DAPI, and a transiently expressed DER-1-GFP plasmid shows co-localization of *Tg*LIPIN to the ER of the parasite. Scale bar: 2.0 μm.

Bioinformatics analysis revealed that *T. gondii* parasites possess a single lipin homolog, much larger than other phosphatidate phosphatases encoded by the genome of *T. gondii*, and which we named *Tg*LIPIN (TGGT1_230690). *Tg*LIPIN possesses the two typical and highly conserved domains of eukaryotic lipins, the amino-terminal N-LIP domain, and the carboxy-terminal C-LIP domain harboring its functional PA phosphatase catalytic motif DXDXT/V (HAD-like domain, Fig. 1a)^17^. Phylogenetic analysis confirms that the enzyme is highly conserved as a single lipin within phylum Apicomplexa, cladding specifically within a coccidian subgroup (Supplementary Fig. 1a).

To confirm the predicted phosphatidic acid phosphatase activity of *Tg*LIPIN (Fig. 1b), we performed heterologous complementation using a *Saccharomyces cerevisiae* triple mutant, *∆dpp1∆lpp1∆pah1*, that is deficient in PAP activity with a temperature-sensitive phenotype (low growth at 37 °C)^18,19^. We attempted to generate the full *Tg*LIPIN sequence and the corresponding recombinant protein but were not able to do so, due to limitations posed by the large size of the protein. Instead, we successfully generated *Tg*LIPIN C-LIP domain, containing the putative catalytic (HAD-like) domain of the protein, and expressed it in the yeast mutant. We thus conducted lipidomic analyses on the yeast mutant, complemented or not with *Tg*LIPIN C-LIP domain. The yeast mutant displayed a severe and significant accumulation of PA and a significant reduction of DAG compared to the wild-type yeast strain, corresponding to the loss of PA phosphatase activity (Fig. 1c). Complementation of the yeast mutant with the C-LIP domain of *Tg*LIPIN fully restored the levels of PA and DAG to those of the wild type, thus confirming its predicted PAP activity (Fig. 1b, c).

To determine its cellular localization, *Tg*LIPIN was endogenously tagged with 3×HA at its C-terminal end^20^. Immunofluorescence assay (IFA) and colocalization with known organelle markers revealed a broad cytosolic and perinuclear localization (Supplementary Fig. 1b). Interestingly *Tg*LIPIN lacks an apparent nuclear localization signal (NLS), possessed by most eukaryotic *LIPINs*^8,21^. To further resolve its localization, we conducted IFAs of *Tg*LIPIN-HA with a known *Toxoplasma* ER marker, DER-1-GFP^22^ an episomally expressing plasmid, which confirmed proximity to the endomembrane system with partial ER colocalization (Fig. 1d). *Tg*LIPIN was not further detected outside the parasite or in the host cell (Supplementary Fig. 1b).

### *Tg*LIPIN disruption induces rapid membrane malformation, division, and replication defects leading to parasite death, all aggravated under a high host nutrient environment

**Fig. 2:**
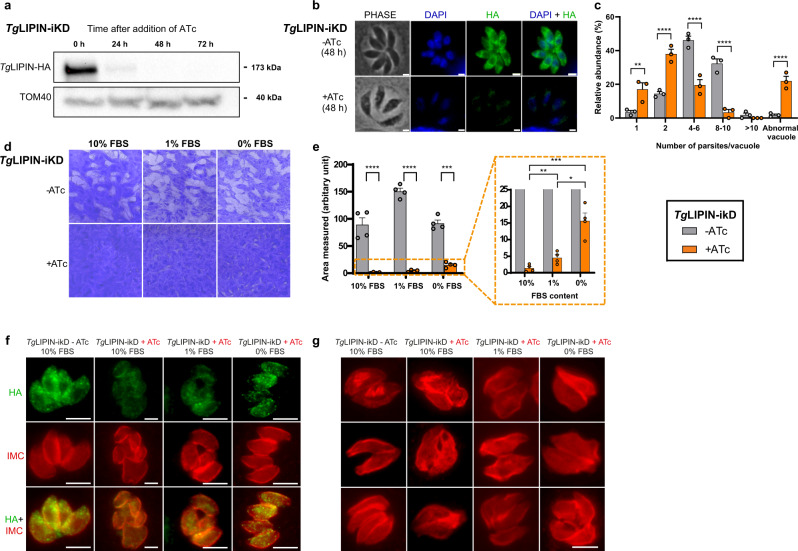
*Tg*LIPIN is indispensable for parasite replication and growth within its host. **a** Western blot shows *Tg*LIPIN downregulation, 48 h + ATc (0.5 μg/mL), *Tg*LIPIN (anti-HA), and TOM40 (control). **b** IFA of *Tg*LIPIN-ikD, indicating loss of protein using anti-HA antibody at 48 h +ATc, Scale bar: 2.0 μm. **c** Replication rate of *Tg*LIPIN parasites grown with (+) or without (−) ATc measured by parasite number per parasitophorous vacuole after 24 h of growth post infection (>100 vacuoles were counted per biological replicate; *n* = 3, unpaired *t* test *P* values where **P = 0.002 −ATc vs +ATc 1 parasite/vacuole, ****P < 0.000001 −ATc vs +ATc 2/4-6/8-10 and abnormal parasite/vacuole). Data are presented as mean values + /− SEM. **d** *Tg*LIPIN-ikD plaque assays measuring parasite growth over 8–10 days (+/−ATc) in different FBS conditions. **e** shown as a bar graph (10%, 1% and 0%; *n* = 4, unpaired *t* test *P* values where ****P < 0.000001 −ATc vs +ATc 10/1/0% FBS, **P = 0.026 +ATc 10% FBS vs +ATc 1% FBS, ***p = 0.0001 +ATc 10%FBS vs +ATc 0% FBS, **P = 0.0047 +ATc 1% FBS vs +ATc 0% FBS). Data are presented as mean values + /− SEM. **f** IFA illustrating early phenotypic effects of *Tg*LIPIN depletion (24 h+ ATc) showing the presence of residual protein (HA-green). Inner membrane complex antibody (IMC- red) clearly shows aberrant IMC membrane biogenesis. **g** IFA illustrating the phenotypic effects of *Tg*LIPIN depletion after 48 h (+ATc) using anti-IMC antibody in *Tg*LIPIN-ikD. Scale bar: 5.0 μm.

To understand the importance of *Tg*LIPIN in parasite growth, we generated an N-terminal HA-tagged inducible knockdown parasite line based on the Tet-off system, *Tg*LIPIN-ikD^23,24^ (Supplementary Fig. 2a, b). Downregulation of *Tg*LIPIN-ikD showed no detectable protein by western blot after 48 h of anhydrotetracycline (ATc) treatment (Fig. 2a). Cytoplasmic localization of the protein in the same manner as C-terminally tagged strains and its downregulation were both confirmed by IFA (Fig. 2b). This result suggests that *Tg*LIPIN disruption dramatically impacts parasite intracellular growth and development *Tg*LIPIN disruption caused a severe intracellular replication defect in *Tg*LIPIN-ikD with a significant increase of small vacuoles containing 1–2 parasite vacuoles (15–20%) and vacuoles containing morphologically abnormal parasites (15–20%), alongside with a concomitant significant decrease of larger vacuoles (25–30%) containing 4–10 parasites (Fig. 2c). To further assess the effect of *Tg*LIPIN on parasite intracellular growth, plaque assays were performed investigating the mutant’s capacity to maintain proper growth with fluctuating levels of host nutrients. When cultured in regular growth conditions with 1% FBS, *Tg*LIPIN-ikD (+ATc) exhibited a severe growth defect with few plaques (Fig. 2d, e) as expected from IFA results (Fig. 2b, f). To rescue this defect, the parasites were grown in high nutrient content with 10% FBS^4^. However, surprisingly, *Tg*LIPIN-ikD (+ATc) showed a more significant decline in growth in 10% than with 1% FBS containing culture medium, as marked by the complete absence of plaques (Fig. 2d, e). Contrastingly, a decrease in the host nutritional environment with 0% FBS slightly but significantly enhanced the growth of *Tg*LIPIN-ikD (+ATc) (Fig. 2d, e) suggesting that exogenous lipid-nutrient source of FFA or PL are somehow toxic to parasites lacking *Tg*LIPIN. To further delineate the division defect phenotype of *Tg*LIPIN downregulation, we performed IFAs to probe the morphology of the inner membrane complex (IMC) as a marker of parasite shape and division. IFAs were conducted prior to and after complete protein loss, at 24 h (Fig. 2f and Supplementary Fig. 2c) and 48 h (Fig. 2g) of ATc treatment, respectively, under low, normal, and high host nutrient contents. Severe membrane anomalies and division defects were observed even as early as 24-h ATc treatment. Such membrane defects were strongly increased at 48-h ATc treatment, with membrane extrusion, loss of parasite integrity, and division arrest. Importantly, IMC and division defect were more evident when host nutrient content increased.

### Electron microscopy reveals gross membrane anomalies as an early impact of *Tg*LIPIN downregulation

**Fig. 3:**
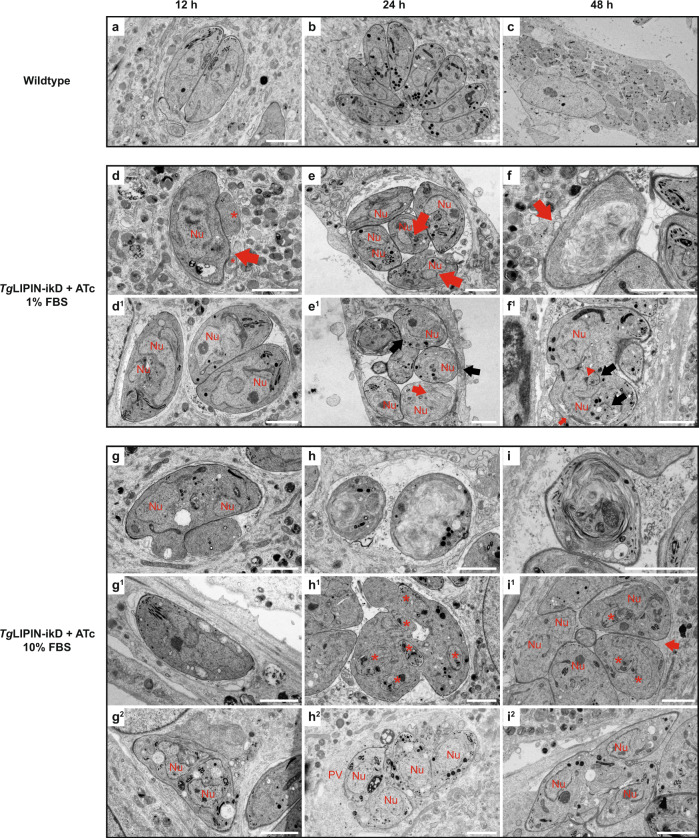
*Tg*LIPIN depletion results in gross membrane anomalies early in the process of ATc downregulation. Transmission electron micrographs showing wild-type (**a**–**c**) and *Tg*LIPIN-ikD parasites at 1% (**d**–**f**) or 10% FBS (**g**–**i**), after 12, 24, and 48 h of ATc treatment. After 12 h of growth, wild-type parasites showed vacuoles containing (**a**) 2–4 parasites, (**b**) 8–16 parasites at 24 h, and (**c**) after 48 h, the first round of egress/reinvasion has occurred showing cells with multiple small vacuoles showing regular nuclei and organelles. **d**–**f** *Tg*LIPIN-ikD parasites show membrane anomalies as early as **d**–**d**^**1**^ 12 h of induction. Vacuoles showed large inclusions in the parasitophorous vacuole, red asterisks. **d**^**1**^ Nuclei showed a multilobed shape even in the absence of early signs of mitosis. **e**–**e**^**1**^ At 24 h, nuclear envelope showed pronounced anomalies with elongations connected with rough ER tubules, red arrows. **e**^**1**^ The ER showed exaggerated proliferation generating big membrane whorls. Incomplete cytokinesis was observed, black arrow. **f** Forty-eight hours after induction, parasites were full of ER-derived membranes and there was the fusion of the parasitophorous membrane (PVM) and parasite plasma membrane (PM), red arrow. **f**^**1**^ Endodyogeny defect: a new round of mitosis started, shown by the presence of IMC buds (black arrows) and centrocone (red arrowhead), while two sister cells from the previous generation have not separated. **g**–**i** All membrane defects were present and enhanced in 10% FBS. **g**–**g**^**1**^ At 12 h, nucleus elongation and cytoplasmic evaginations were observed. **g**^**2**^ Cytokinesis failure was observed. **h**–**h**^**2**^ At 24 h, ER proliferation and cytokinesis defect were observed. Vacuoles showed signs of necrosis with PM rupture and cytoplasmic leakage in the vacuolar space. **i** At 48 h, *Tg*LIPIN-ikD did not reinvade, the remaining vacuoles showed profoundly abnormal parasites, **i**^**1**^ PVM or **i**^**2**^ PM rupture/cytokinesis failure. Nu nucleus. Scale bar: 2 µm, *n* = 3.

To further resolve the cellular phenotype marked by *Tg*LIPIN downregulation, *Tg*LIPIN-ikD (+ATc) parasites were examined by transmission electron microscopy (TEM) at 12, 24, and 48 h post ATc treatment under low, normal, and high host nutrient content (0%, 1%, and 10% FBS, respectively). Interestingly, as early as 12 h of treatment with ATc, when *Tg*LIPIN levels are only slightly reduced, both parasite IMC and plasma membrane displayed gross abnormalities in forming evaginations or lateral interruptions, without affecting the parasite size (Fig. 3a–c). Concordantly, IFA data of *Tg*LIPIN-ikD (+ATc) also hinted at aberrant IMC (Fig. 2f, g). The morphology of the nuclear envelope (NE) started to adopt tubular or multilobed shapes (Fig. 3d–d^1^ and Supplementary Fig. 3). Extensions of the NE seemed to expand in the cytoplasm in connection with the ER (Supplementary Fig. 3a) making tubular whorls in the cytoplasm (Fig. 3e–e^1^). At 48-h treatment, tubular or multilamellar membrane accumulation could entirely fill the cytoplasm of the parasites (Fig. 3f–f^1^). Local fusion of the outer membrane of different nuclear lobes could be observed (Supplementary Fig. 3b). The NE tended to adopt hairpin shapes where the inner membrane from two different regions was seen closely apposed, separated by electron-dense material (Supplementary Fig. 3c, e). Local detachment and fusion of the inner membranes could be also observed (Supplementary Fig. 3e, red arrow). Interestingly, similar expansions affecting the NE are also observed in the yeast LIPIN mutant coupling phospholipid biosynthesis to the nuclear membrane^25,26^.

Both parasite IMC and plasma membrane (PM) also displayed abnormalities in forming bumps (Fig. 3d, red arrow) or lateral evaginations (Fig. 3g–g^2^). Large inclusions could be observed in the vacuolar space that could correspond to such abnormal parasite evagination depending on the cutting plane. Complex structures were also observed in the cytoplasm, showing lasso-like structures formed by zippered membrane tubules (Supplementary Fig. 3f, g). It is not clear whether this type of structure originated from the ER, the nuclear membrane, or even IMC.

At 48 h, the ultrastructure of the vacuole appeared dramatically altered. Endodyogeny was strongly affected in *Tg*LIPIN-ikD parasites, showing incomplete sister cells separation (Fig. 3e^1^–f^1^), cytokinesis failure harboring multiple daughter cells (Fig. 3h–i^2^, red asterisk). Local fusion of the parasitophorous membrane (PVM) with the PM of the parasite was also observed (Fig. 3f). Some vacuoles showed signs of parasite PM rupture, with cytoplasmic material reminiscent of parasite cytoplasm filling the vacuolar space (Fig. 3g^2^, h^2^, i^1^). Large autophagosomes with unidentified content were also frequently observed in infected host cells suggesting that the parasites died and were eliminated by the host cell (Supplementary Fig. 3h–k). Large giant multivesicular bodies (gMVBs) and swelling of the host NE, previously described as enhanced under starvation conditions^4^ were abundant in *Tg*LIPIN-ikD parasites, even in the presence of 10% FBS. In some cases, the outer membrane of the NE had fused with the PVM (Supplementary Fig. 3c), occasionally leading to its rupture (Supplementary Fig. 3d).

In agreement with the replication assay data (Fig. 2c), TEM images showed that dynamics of parasite membranes was profoundly affected by the absence of appropriate levels of *Tg*LIPIN, which seemed to start at the NE level as soon as 12 h of treatment. Further loss of *Tg*LIPIN then induced strong aberrant membrane abnormalities mostly affecting the endomembrane system (NE, ER, IMC, and PM). This resulted in endodyogeny failure, anarchic membrane proliferation, and unexpected membrane fusion events, leading to parasite growth arrest and death. These membrane abnormalities and division phenotypes were observed in all host nutrient contents, but were always stronger and aggravated under high host nutrient content (10% FBS). This suggests that the essential role of *Tg*LIPIN for membrane biogenesis and parasite survival is directly linked to the nutritional status of the host.

### *Tg*LIPIN regulates lipid homeostasis

**Fig. 4:**
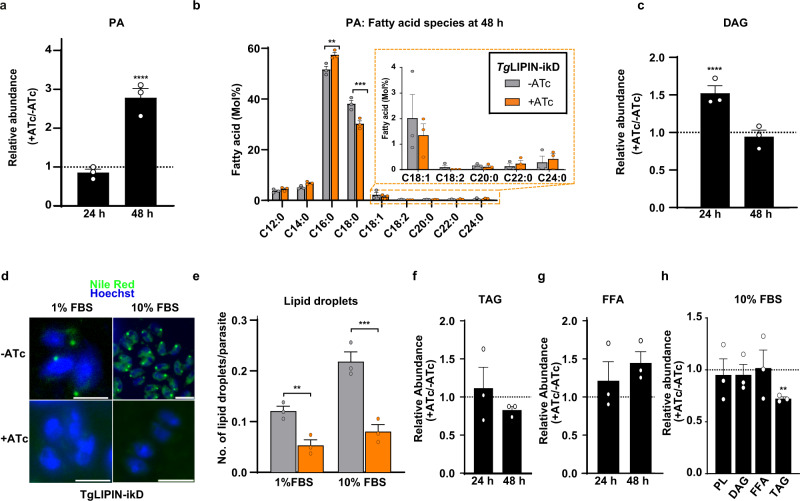
*Tg*LIPIN regulates critical levels of PA and other major phospholipids while generating DAG that is directed toward neutral lipid storage. **a** Relative abundance of PA in *Tg*LIPIN-ikD (nmol/parasites number, +ATc/−ATc; %; *n* = 3, unpaired *t* test *P* values where ****P < 0.00001 relative PA abundance ratio nmol/parasites number +ATc/−ATc at 48 h vs +ATc/−ATc = 1). **b** Fatty acid composition of PA in *Tg*LIPIN-ikD (+/−ATc) in Mol%; *n* = 3, unpaired *t* test *P* values where **P < 0.0021 PA C16:0 −ATc vs +ATc, ***P < 0.0002 PA C18:0 −ATc vs +ATc. **c** Relative abundance of DAG (+ ATc/−ATc) is reduced by *Tg*LIPIN depletion after 48 h + ATc (*n* = 3, unpaired *t* test *P* values where ****P < 0.00001 relative DAG abundance ratio nmol/parasites number +ATc/−ATc at 24 h vs +ATc/−ATc = 1). **d** Nile red staining showed the number of lipid droplet within the parasites after 24 h of + /−ATc (scale bar = 5.0 μm) with (**e**) a representative bar graph (*n* = 3, unpaired *t* test *P* values where **P < 0.0093 number of lipid dro*p*lets per parasite +ATc/−ATc at 1% FBS, ***P < 0.0003 at 10% FBS). **f** Relative abundance of TAGs in *Tg*LIPIN-ikD (+ATc/−ATc) showing that TAG levels were relatively reduced in the *Tg*LIPIN-ikD (48 h + ATc). **g** Relative abundance of free fatty acids (FFA, +ATc/−ATc in the *Tg*LIPIN-ikD). **h** Relative abundance of PLs, DAG, FFA and TAG in the *Tg*LIPIN-ikD +ATc to −ATc at 10% FBS (*n* = 3, unpaired *t* test *P* values where **P < 0.004 relative TAG abundance ratio nmol/parasites number +ATc/−ATc at 10% FBS vs +ATc/−ATc = 1). Data are presented as mean values + /− SEM.

To determine the functional role of *Tg*LIPIN in parasite PA synthesis and membrane biogenesis, we conducted comprehensive lipidomic analyses. To precisely assess the timely impact of *Tg*LIPIN-ikD disruption on lipid synthesis, parasites were grown for 24 or 48 h with and without ATc. Total lipids were separated by high-performance thin-layer chromatography (HPTLC) and quantified by gas chromatography–mass spectrometry (GC–MS). Relative abundance of PA was unchanged at 24 h of +ATc (Fig. 4a). After 48 h +ATc, there was a large significant increase of PA, confirming its role for PA synthesis and our WB results for the loss of TgLipin (Fig. 4a). FA composition of PA showed an increase in the level of myristic (C14:0) and palmitic (C16:0) acids along with a decline in the levels of stearate (C18:0) (Fig. 4b). Concomitantly to PA increase, the relative abundance of DAG was decreased after 48 h of *Tg*LIPIN downregulation by ATc (Fig. 4c) showing that *Tg*LIPIN is an active phosphatidic acid phosphatase, controlling the levels of PA and DAG, as confirmed by our yeast heterologous complementation (Fig. 1c). Despite a strong replication defect and reduction in parasite number, the total amount of phospholipids was increased up to 1.5-fold in the *Tg*LIPIN-ikD (+ATc) in comparison to the control (−ATc) (Supplementary Fig. 4a). Accordingly, most PL classes were further increased by *Tg*LIPIN disruption such as the ones usually made from PA via the CDP–DAG pathway^25^ i.e., cardiolipin (CL), PS, and PC (Supplementary Fig. 4b). This suggests that *Tg*LIPIN controls PL homeostasis likely through PA synthesis.

In eukaryotes, TAG biosynthesis occurs at the last step of the glycerol-3-phosphate pathway through the *sn-3* acylation of DAG catalyzed by a diacylglycerol-acyltransferase (DGAT) that uses FFA^7,26,27^. However, the key and limiting step of TAG synthesis is the formation of its precursor, DAG, the production of which depends on the action of a PAP/LIPIN. TAG is not a membrane but a storage lipid that accumulates within lipid droplets together with other neutral lipids such as cholesteryl esters (CE). We thus investigated the putative role of *Tg*LIPIN in lipid-storage formation by comparing the number of parasite lipid droplets using nile red staining^28^ in the *Tg*LIPIN-ikD parasites (+ATc and −ATc). This showed that the number of lipid droplets per parasite vacuole in the presence of ATc was reduced to almost 60% in comparison to the control in both normal (1% FBS) and furthermore in high host nutrient content (10% FBS) (Fig. 4d, e), suggesting that *Tg*LIPIN is involved in the synthesis of storage lipids. According to this hypothesis, the relative abundance of TAGs was significantly reduced after 48 h of ATc treatment (Fig. 4f). Other neutral lipids making the bulk of lipid droplets, CE levels were also significantly decreased (Supplementary Fig. 4d).

TAGs and CEs are neutral storage lipids potentially implicit in the parasite’s ability to cope with the excess FA^7^. The synthesis of TAG via enzyme DGAT requires two substrates, DAG and FFA. We, therefore, determined the FFA content of the *Tg*LIPIN-ikD. Lipidomics revealed slight increases in total FFA levels in the *Tg*LIPIN-ikD (+ATc/−ATc) (Fig. 4g). Interestingly, C18:1 (oleate) was the only FA found significantly increased in the parasite-free FA (FFA) pool (Supplementary Fig. 4c), suggesting C18:1 may be an important intermediate in the pathways regulated by *Tg*LIPIN, as previously hinted^7,29,30^. In a high host environment (10% FBS), lipidomic analyses revealed that only TAG levels decreased under these conditions (Fig. 4h), explaining the reduction of lipid droplets. This impairment in TAG biosynthesis is consistent with the reduction in parasite viability seen under high FBS conditions (Fig. 2d, e).

### *Tg*LIPIN channels the flux of host fatty acids to prevent the accumulation of free fatty acids levels and promotes TAG synthesis

Our data reveal the essential role for *Tg*LIPIN in regulating PA and DAG synthesis to control lipid homeostasis, especially the level of storage lipid molecule, TAG. *Tg*LIPIN function seems directly connected to host nutritional status and the origin of lipid precursors used (FASII, host cell, and external environment) to maintain parasite survival.

**Fig. 5:**
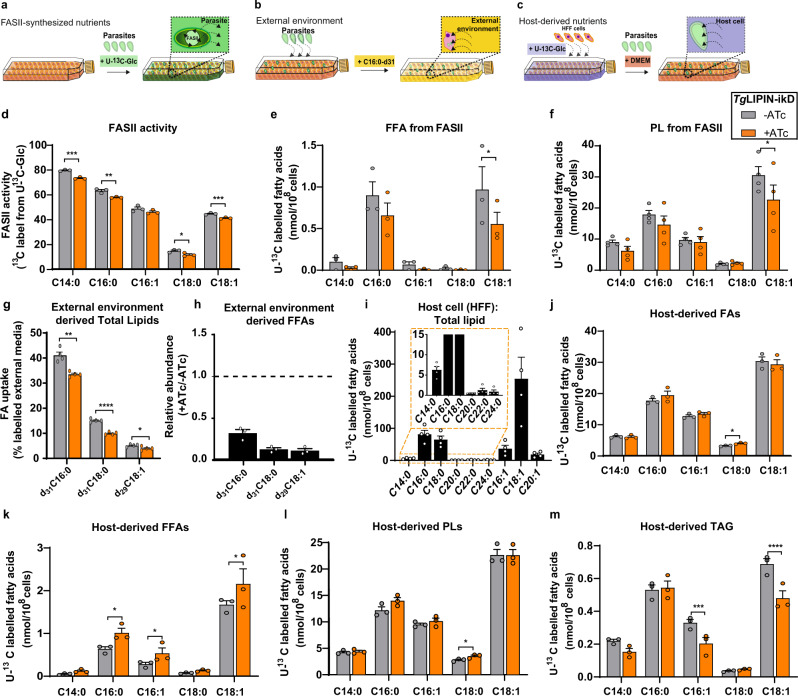
Monitoring the source of fatty acids in *Tg*LIPIN-ikD. **a**–**c** Schematic for the experimental procedure of stable isotope labeling to determine the origin of fatty acids in *Tg*LIPIN-ikD; **a** Parasite FASII derived FAs were determined by the addition of U-^13^C-glucose (Glc) to the culture medium (1% FBS, no glucose, +/−ATc) at the time of parasite infection to the host. **b** FAs scavenged from the external environment (medium) was determined by the addition of ^31^d-palmitic acid (^31^d-C16:0) to the parasite culture medium (high glucose, 1% FBS). **c** Host cell-derived FAs were determined by growing host cells in the presence of U-^13^C-glucose (no glucose, 10% FBS) to confluent in prior to the infection parasites with normal medium (glucose, 1% FBS, + /−ATc). **d** FASII activity in *Tg*LIPIN-ikD as measured as % ^13^C-incorporation (*n* = 3, unpaired *t* test *P* values; ***P < 0.0005% FASII derived C14:0/C16:0/C18:0/C18:1 −ATc vs +ATc, **P < 0.007/*P < 0.04/***P < 0.0003). **e** Abundance of apicoplast FASII derived ^13^C labeled FFAs (*n* = 3, unpaired *t* test *P* values; *P < 0.02 of FASII derived FFA C18:1-ATc vs +ATc). **f** Abundance of apicoplast FASII derived ^13^C-labeled phospholipids (PLs, *n* = 4, unpaired *t* test *P* values; *P < 0.03 of FASII derived PL C18:0-ATc vs +ATc). **g** FA uptake of deuterated external environment-derived FAs (*n* = 4, unpaired *t* test *P* values; **P = 0.0034/***P < 0.00001/*P = 0.01 FA uptake from the external environment as d_31_C16:0/d_31_C18:0/d_29_C18:1 −ATc vs +ATc). **h** Relative abundance of deuterated external environment-derived (+ATc/−ATc) (*n* = 3). **i** The abundance of ^13^C-labeled fatty acids in the host cells (*n* = 4). **j** Host derived FAs measured as % ^13^C-incorporation (*n* = 3, unpaired *t* test *P* values; *P < 0.02 Host derived FA C18:1-ATc vs +ATc). **k** Abundance of host-derived ^13^C-labeled FFAs (*n* = 3, unpaired *t* test *P* values; *P < 0.05 Host derived FA C16:0/16:1/18:1−ATc vs +ATc). **l** Abundance of host-derived ^13^C labeled PL (*n* = 3, unpaired *t* test *P* values; *P < 0.023 Host derived PL C18:0−ATc vs +ATc) and **m** TAG levels (*n* = 3, unpaired *t* test *P* values; ***P < 0.0001 Host-derived TAG C16:0/18:1−ATc vs +ATc). Data are all presented as mean values + /− SEM.

Fatty acids required for lipid synthesis in *T. gondii* parasites are obtained from the host and/or host environment (i.e., medium)^1,2,4,26,29^ and/or synthesized de novo via the apicoplast FASII pathway^12,30,31^. To identify the source of increased FA and phospholipids detected upon *Tg*LIPIN depletion, we set up fluxomics approaches where we grew parasites and/or host cells using different stable isotope substrates-containing media (^13^C-U-glucose, ^31^d-C16:0) and performed lipidomic analyses for each experimental setup (Fig. 5a–c).

To monitor apicoplast FASII activity, parasites were labeled with U-^13^C-glucose, which was added to a glucose-free medium, to confluent host cells together with parasites with or without ATc (Fig. 5a), as previously reported^13^. Total lipid content, PL, and neutral lipids were extracted to determine the ratio of ^13^C incorporation of each FA as a measure of FASII activity. In *Tg*LIPIN-ikD (+ATc), the amount of labeled FA made by FASII (i.e., FASII activity) decreased under the suppression of *Tg*LIPIN (+ATc) in terms of total lipid content (Fig. 5d) as well as the pools of FFA/PL/TAG (Fig. 5e, f and Supplementary Fig. 5a, b). Interestingly, there was a significant decrease in all the signature FA from FASII (i.e., C14:0) but also in FASII-derived FA, i.e., oleic acid C18:1, reduced in total lipids, FFA, PL and TAG made from FASII (Fig. 5d, e, f and Supplementary Fig. 5a, b). There was an overall decrease in FASII-derived C18:1 in FFA (Fig. 5e) and TAG (Fig. 5f, supporting the lipid droplet analyses) and an increase in PC16:0 (Supplementary Fig. 5b). This suggests that with the loss of *Tg*LIPIN, the increased overall PA levels with FASII-derived FAs, can be directed towards the production of excess PC. The distribution of ^13^C incorporation to each isotopologue of FASII FA product confirmed that the pathway was nevertheless functional (Supplementary Fig. 5c–g). To monitor the FAs sourced from the external environment/culture medium, we grew parasites and confluent host cells in a medium supplemented with deuterated palmitic acid (^31^d-C16:0) (Fig. 5b). Parasites were harvested and total lipids were extracted to determine the incorporation of ^31^d-C16:0 in parasite lipids. In the total lipid content, ^31^d-C16:0 and its elongation/desaturation products ^31^d-C18:0 and ^29^d-C18:1 were detected in lower amounts in the *Tg*LIPIN-ikD parasites with ATc (Fig. 5g). In addition, a drastic reduction in the incorporation of all three (^31^d-C16:0, ^31^d-C18:0, ^29^d-C18:1) to FFA species within *Tg*LIPIN-ikD (+ATc) in comparison to the control (−ATc), further suggesting that the host external environment is not the source of increased lipids within the mutant (Fig. 5h).

To monitor the fatty acids directly scavenged from the host, we designed a novel assay using U-^13^C-glucose to label host lipids (Fig. 5c). Nonconfluent host cells were grown in the presence of medium containing U-^13^C-glucose which would theoretically fuel any of the active FA synthesis pathways within the host (FASI, elongases) via the synthesis of their substrates, acetyl-CoA, thereby generating ^13^C-pre-labeled host metabolites including lipids and fatty acids^32^. These ^13^C-pre-labeled host cells were then infected with parasites in the presence of a normal culture medium, which contains regular ^12^C-glucose. Total lipid was extracted from the parasites to determine the ratio of ^13^C incorporation to each fatty acid and determine the origin from the host or not. Using this novel approach, we were able to determine (i) the FA biosynthetic capacities of the host cells (Fig. 5i), and more importantly (ii) the first scavenged FA lipidome of the parasite (Fig. 5j–m), the scavengome. Host cells, human foreskin fibroblast (HFF), are capable of synthesizing FA ranging from C14:0 to C20:1, with the most abundant FA species being C18:1 (Fig. 5i). The wild type (*Tg*LIPIN-ikD −ATc) parasites are capable of scavenging all these FA species made by the host cell with a major preference again for C18:1 (Fig. 5j). Importantly, in *Tg*LIPIN mutant (+ATc), ^13^C-labeled C16:0, C18:0 and C18:1 from host cells were significantly increased in the FFAs (Fig. 5k), together with a significant increase of host-derived C18:0 in both parasite total lipids and PL (Fig. 5j, l). Therefore, since FA from both FASII and the external environment decrease in the absence of *Tg*LIPIN, whilst FAs from host cell are found increasing, our data strongly suggest that the major origin of excess lipids in the *Tg*LIPIN mutant is from the host cell. Correlating with the lipid droplet analyses, there was a significant decrease in the ^13^C labeled FA, specifically C16:1 and C18:1, in TAG (Fig. 5m). The distribution of ^13^C incorporation to each isotopologue of host-derived FAs was also determined (Supplementary Fig. 5h–m). Together these data show that the disruption of *Tg*LIPIN induced the massive accumulation of host cell-scavenged FA, specifically including C18:1, that cannot be used for TAG synthesis.

Hence, *Tg*LIPIN regulates parasite membrane biogenesis by controlling the synthesis and content of PA and DAG, and by channeling the constant flux of host cell scavenged FA for TAG. Without *Tg*LIPIN, the parasite accumulates toxic levels of PA, FFA, and PL that eventually cause massive membrane malformations, cytokinesis defect, and parasite death.

## Discussion

**Fig. 6:**
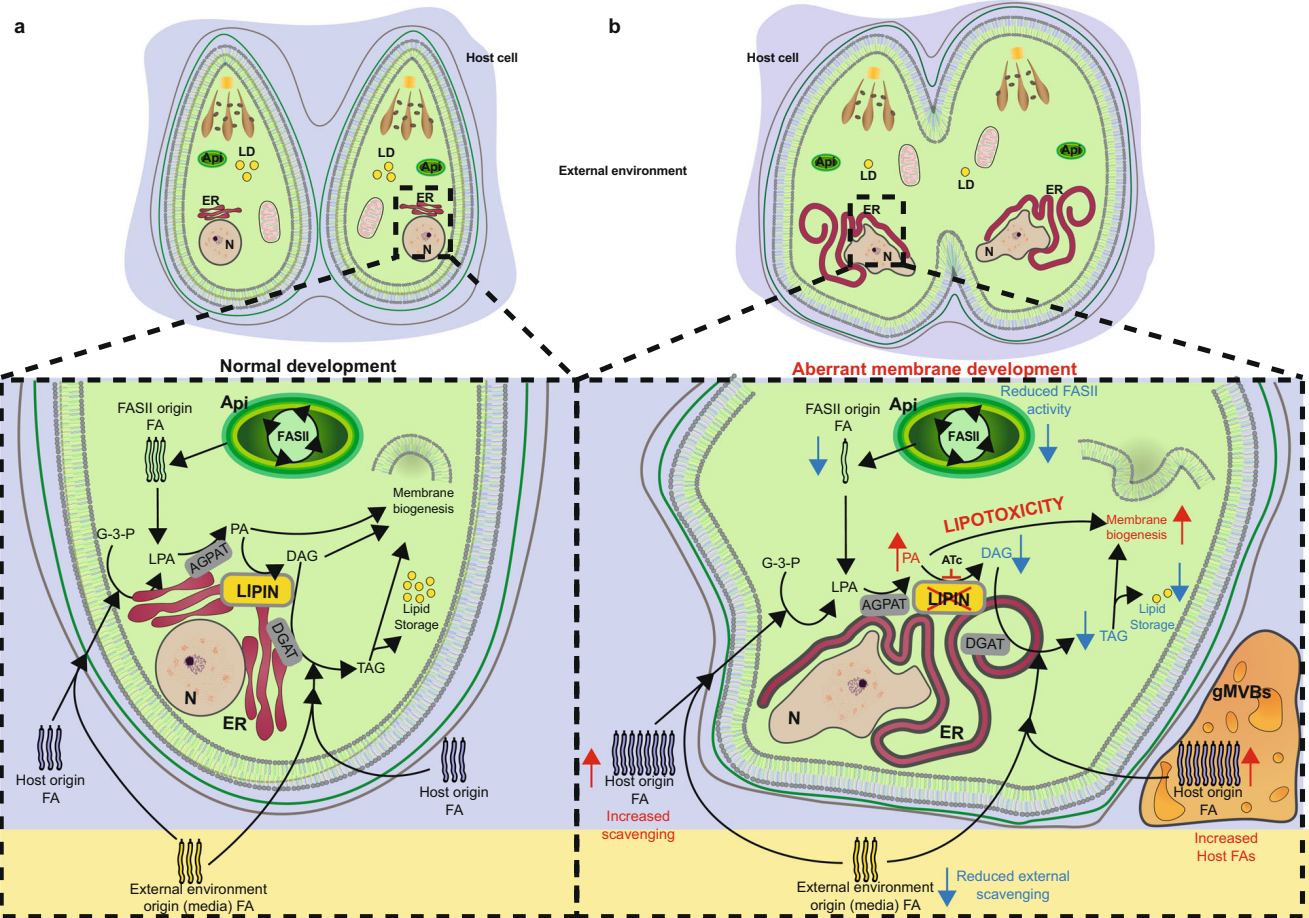
Proposed role of LIPIN in *Toxoplasma* lipid metabolism. Schematic representation of the dual essential role of *Tg*LIPIN in parasite lipid metabolism, including, membrane biogenesis, and lipid storage. **a** The parasite utilizes, and glycerol-3-phosphate (G-3-P) and fatty acid (FA)s derived from both apicoplast (Api) FASII and host to synthesize major lipids by forming lysophosphatidic acid (LPA) followed by phosphatidic acid (PA). PA is hydrolyzed by *Tg*LIPIN to generate diacylglycerol (DAG), which is further acylated to generate triacylglycerol (TAG). The other branch of this pathway redirects PA toward the generation of major membrane phospholipids. The FA homeostasis between membrane biogenesis and storage essential for normal parasite development within its host is maintained by the PA phosphatase *Tg*LIPIN. **b** Genetic ablation of *Tg*LIPIN in *Tg*LIPIN-ikD parasites in the presence of anhydrotetracycline (ATc) caused the PA/DAG imbalance. The increased PA is channeled towards PL synthesis resulting in excess PLS and consequent gross membrane anomalies within the parasite IMC, nucleus, and ER, while forming giant multivesicular bodies (gMVBs). Simultaneously, the reduction in DAG affected TAG biogenesis and hence the lipid storage capacity, marked by decreased lipid droplets (LD), within the parasite lacking *Tg*LIPIN. The impairment of TAG biosynthesis, resulted in excess fatty acids derived from the host, within *Tg*LIPIN mutant, causing lipotoxicity.

*Tg*LIPIN plays a pivotal role in the synthesis of central lipid precursors, PA and DAG while regulating the flux of host lipids thereby controlling the equilibrium between membrane biogenesis and lipid storage. Collectively this controls intracellular development and the propagation of the parasite (Fig. 6a).

Our data here show that *Tg*LIPIN is an essential PAP controlling the PA/DAG equilibrium along with the level of FFAs. Disruption of this equilibrium together with impairment of storage capacity has multiple phenotypic consequences within the parasite (Fig. 6b). In mammalian cells, LIPIN is encoded by three independent genes *lipin*1, 2, 3; all of which have PAP activity modulating the levels of TAGs and phospholipids^21^. Genetic mutation within these LIPINs causes severe metabolic disorders, including rhabdomyolysis (*lipin*1), obesity (*lipin*1), autoinflammatory disease (*lipin*2), and impaired lipoprotein assembly in the intestines (*lipin*2 and 3)^33^. The essentiality of LIPIN for *T. gondii* growth also suggests distinct functionality from the two other parasite PAPs. Neither *Toxoplasma* PAPs appeared to compensate for the loss of *Tg*LIPIN, unlike in yeast whereby deletion of genes encoding LIPIN can be partly compensated by other PAPs^19^. The absence of a NLS (nuclear localization signal) in the *Tg*LIPIN sequence is consistent with a cytosolic localization, colocalization specifically with the NE membrane, ER, and other endomembrane. The localization and catalytic activity of LIPIN proteins in humans and yeast are regulated by a phosphatase bearing the HAD domain as its substrate^34–36^.

Therefore, it is conceivable that *Tg*LIPIN is quite dynamic and can be recruited on endosomal membranes to regulate the PA/DAG equilibrium, depending on metabolic demand, cell cycle, or coupled to membrane trafficking events. This hints at a probable link between metabolism and the nucleus of the parasite, however contrary to yeast and mammals, there is no evidence of a direct effect of *Tg*LIPIN on gene regulation, which could explain the absence of nuclear localization.

One of the early phenotypic stresses evoked in the *Tg*LIPIN-deficient mutant was the impact on major membranes including the nuclear envelope complexed with the ER and the parasite IMC. There is evidence that suggests that nuclear membrane biogenesis is linked to PA metabolism^37,38^. In *∆pah1* yeast, mutations in upstream biosynthetic steps of the glycerolipid pathway that lower PA levels also reduced the aberrant expansion of nuclear membrane^37^. Effects of *pah1* deletion could be phenocopied by overexpression of Dgk1p, a nuclear/ER membrane DAG kinase that generates PA^37^.

A recent study demonstrated that the use of DGAT inhibitor-T863, inhibiting DAG to TAG synthetic reaction in *T. gondii* parasites resulted in rapid accumulation of membranous structures accompanied by rapid parasite death due to strong replication defect^6^. This cytopathy was restricted mainly to the ER and the associated nuclear envelope, similarly as in the case of *Tg*LIPIN depletion. The electron micrographs of the *Tg*LIPIN mutant (+ATc) clearly suggest that the replication arrest was initially due to profound modifications of the nuclear membrane resulting in aberrant membrane proliferation, cytokinesis defect, and parasite death.

In the absence of *Tg*LIPIN, we observed an overall increase in the major membrane phospholipids, including CL, PS, and PC which is a derivative of the CDP-DAG pathway in other eukaryotes^39^. In absence of DAG, PL biosynthesis was expected to be reduced. Instead, an increase in both PL and FFA is observed, which is likely due to excessive host lipid scavenging. The TAG content in *Tg*LIPIN mutant decreases only 48 h post protein downregulation with ATc suggesting that due to a decrease in the substrate-specific DAG species because of *Tg*LIPIN unavailability, the parasite is unable to acylate the free fatty acids to generate its subsequent product TAG. This is supported by the drop in lipid droplet numbers in 1% and 10% FBS when *Tg*LIPIN is suppressed and the exaggerated effect in a high host nutrient environment (Fig. 4d, e). In mammalian cells the inhibition of TAG synthesis leads to oleic acid (C18:1) induced lipotoxicity^39^. Thus, lipid droplets act as more than just cellular energy reserves by protecting the cells from deleterious effects of toxic excess FFAs by their incorporation into TAGs.

The prelabeling of host fibroblast cells with U-^13^C-Glucose, allowed us to track the source of excess fatty acid and phospholipid content in the *Tg*LIPIN mutant. In keeping with previously reported data, using ^13^C-fluxomics we observed that the parasite has a specific preference for scavenging oleate (C18:1) directly from host^40^. Existing literature provides strong evidence of the involvement of C18:1 in TAG biosynthesis and subsequent lipid droplet formation in mammals^39^ as well as parasites^7^. Thus, the excess oleate in the form of FFAs within the parasite sourced directly from the host has a strong correlation to the lipid storage defect in *Tg*LIPIN mutant (Fig. 5k). The role of *Tg*LIPIN in the production of TAG was further strengthened by the decrease of host-derived C18:1 in TAG (Fig. 5m). In *T. gondii* parasites, excessive scavenging of C18:1 results in lipotoxicity and parasite death^7^. We show in the absence of *Tg*LIPIN, C18:1 accumulates as FFA and cannot be incorporated into TAG. A significant decrease of C18:1 derived from FASII in the form of FFA is also observed. By sensing this change in nutrients, the parasite attempts to compensate by arresting FASII activity. C18:1 is likely a major FA that is typically scavenged and cannot be stored to avoid lipotoxicity in the absence of *Tg*LIPIN. Taken together, this correlates to previous findings^4^ showing that the parasite is capable of sensing host conditions and metabolically adapt. Similar observations on metabolic plasticity show that there is a reduction in the growth of *P. falciparum* FASII KO mutant in the presence of nutrient-deprived culture conditions^4^. Another study in bacteria shows that the FASII pathway is repressed in a negative feedback loop mediated by acyl-CoA^41^. The mechanisms of metabolic adaptation in Apicomplexa still need to be uncovered. In this study, we were able to reveal the first parasite lipid scavengome, which shows that *Tg*LIPIN prevents parasite ‘lipotoxicity’ in high nutrient environments through effective channeling of host-scavenged fatty acids while maintaining parasite membrane integrity.

## Methods

### Sequence analysis

*Tg*LIPIN (TGGT1_230690) was identified using EuPathDB web sources ToxoDB (http://toxodb.org/toxo/). A phylogenetic tree of LIPIN proteins in several eukaryotes was created using the online platform Phylogeny.fr. The organisms used for LIPIN protein sequences for generation of the phylogenetic tree include *Toxoplasma gondii* (TGGT1_230690), *Plasmodium falciparum* (PF3D7_0303200), *P. berghei* (PBANKA_040180), *Homo sapiens* (NX_Q14693), *H. sapiens* (NX_Q92539), *H. sapiens* **(**NX_Q9BQK8), *Saccharomyces cerevisiae* _PAH1 (PAP1, SMP2, YMR165C, YM8520.14C), *Cryptosporidium parvum* (cgd3_3210), *Cyanidioschyzon merolae* (CYME_CMN061C), *Neospora caninum* (BN1204), *Hammondia hammondi* (HHA_230690), *Chlamydomonas reinhardtii* (CHLRE_12g506600v5), *Arabidopsis thaliana*_AtPAH1 (At3g09560), *Arabidopsis thaliana*_AtPAH2 (At5g42870), *Leishmania major* (LMJF_06_0830), *Trypanosoma brucei* (Tb927.7.5450), *Chromera velia* (Cvel_24403). The first step involved the curation of these protein sequences. The protein sequences were aligned (ClustalW) and then gaps were removed from the alignment. Finally, the phylogenetic tree was constructed using the maximum likelihood method in the PhyML program (3.1/3.0 aLRT). The default substitution model (WAG) was selected. Graphical representation and edition of the phylogenetic tree were performed with Cladogram.

### *T. gondii* strains and cultures

The parasite host cells human foreskin fibroblasts (HFF) were cultured using Dulbecco’s Modified Eagle’s Medium (DMEM, Gibco) supplemented with 10% fetal bovine serum (FBS, Gibco), 2 mM glutamine (Gibco), and 25 μg/mL gentamicin (Gibco) at 37 °C and 5% CO_2_.

*T. gondii* tachyzoite parental strains RH-ΔKu80 TATi, RH-ΔKu80 as well as mutant strains *Tg*LIPIN-ikD, *Tg*LIPIN-3XHA were propagated by serial passage within their host HFF using DMEM supplemented with 1% fetal bovine serum (FBS, Gibco), 2 mM glutamine (Gibco), and 25 μg/mL gentamicin (Gibco) at 37 °C and 5% CO_2_.

### Generation of HA-tagged and inducible knockdown line for *Tg*LIPIN

A C-terminally tagged HA line was generated expressing from the gene’s endogenous locus using the classical pLIC strategy using homologous recombination in a RH-ΔKu80 strain. For the same, a 1677-bp homology region of *Tg*LIPIN located toward to C-terminus excluding the stop codon, was amplified from the parasite genomic DNA using the primers forward 5′-TACTTCCAATCCAATTTAATGCACGGCAGATTTCTCTTACTGG-3′ and reverse 5′-TCCTCCACTTCCAATTTTAGCCAAATTACTGCATTTGCGTTCAC-3′. The homology region was assembled into *PacI* digested pLIC-HA-DHFR plasmid using ligation-independent cloning protocol^17^. The assembled plasmid with linearized with single-enzyme site specific to the parasite DNA sequence within the plasmid-*NsiI* just before transfection. Parasites were selected with the drug pyrimethamine and cloned by limiting dilution.

For generation of inducible knockdown plasmid pPR2-DHFR^42^, two separate homology flanks were chosen. The 5′ flank was amplified 1637 bp upstream of the *Tg*LIPIN start codon using the primers F1-3′ and R1. The PCR product was ligated to *PacI* and *NdeI* digested vector pPR2 using NEB assembly reaction. Next, the 3′ flank was amplified as a 1739-bp fragment beginning at the start codon of *Tg*LIPIN with the primers F2 and reverse R2. The 3′ homology flank was annealed to *XmaI* and *NotI* digested pPR-HA3-DHFR vector that already contained the *Tg*LIPIN 5′ flank. The final cloned vector positions the start codon of *Tg*LIPIN downstream of the ATc-regulatable t7s4 promoter and a 3xHA tag. The resulting vector with *Not*I and transfected this into TATiΔku80 parasites. Parasites were selected with the drug pyrimethamine and cloned by limiting dilution.

Screening of parasite clones where the t7s4 promoter had successfully replaced the native *Tg*LIPIN promoter, was done using the screen primers 1–6 in the combinations described in Supplementary Fig. S2. All PCRs were performed with TaKara primestar max polymerase. The knockdown of *Tg*LIPIN was induced with 0.5 μg ml^−1^ of anhydrotetracycline (ATc). Primer list can be found in Supplementary Table 1.

### Immunofluorescence assay

Primary antibodies anti-HA (Rat, Roche 1:500), anti-IMC1 (mouse, 1:1000), anti-SAG1 (mouse, 1:1000), anti-MIC2 (rabbit, 1:1000), anti-CPN60 (rabbit, 1:1000), and anti-TOM40 (rabbit, 1:1000) were used at dilutions their respective dilutions. Secondary AlexaFluor 488- and 546-conjugated anti-rat, anti-mouse, and anti-rabbit antibodies (Life Technologies) were used at 1/2500. For the immunofluorescence assay (IFA), parasites were grown on confluent HFF on coverslips and fixed in PBS containing 2.5% paraformaldehyde (PFA) for 15 min at room temperature (RT). Samples were permeabilized with 0.25% Triton X-100 in PBS for 10 min at RT prior to blocking in PBS containing 3% BSA and subsequent incubation with primary antibodies then secondary antibodies diluted in the blocking solution. Labeled parasites were stained with Hoechst 33342 (1:10000, Life Technologies) for 20 min and then washed three times in PBS before final mounting of the coverslips on a glass slide using fluorogel. The IFA slides were visualized using fluorescence microscope (Axio Imager 2_apotome; ZEISS, ×60–100 magnification).

### Western blot analysis

Parasites were harvested for western blot analyses after complete egress from their host. To remove any host cell debris, the parasites were passed through a 3-μm filter, then counted by haemocytometer and solubilized in SDS buffer at equivalent cell densities. Equal amount of protein was separated on a 4–12% gradient SDS-polyacrylamide (Life Technologies) and transferred to a nitrocellulose membrane using the XCellII Blot Module (Invitrogen). Primary antibodies anti-HA (rat, Roche) and anti-TOM40^43^ (rabbit) were used at a dilution of 1:500 and 1:1000, respectively. Secondary goat anti-mouse and anti-rabbit horseradish peroxidase (HRP) conjugated antibodies (Thermo Scientific) were used at 1:2000. Protein signal was detected by chemiluminescence after membrane staining with luminata crescendo western HRP detection kit (Millipore). The signal strength of protein was quantified using a BioRad chemidoc imager (BioRad).

### Phenotypic analysis

*Plaque assay*: The extracellular parasites were harvested after filtration and counted by haemocytometer. Then ~500 parasites were inoculated to a confluent HFF flask (25 cm^2^). *Tg*LIPIN-ikD was grown for plaque assay in the presence or absence of Anhydrotetracycline (ATc) (0.5 μg ml^−1^) for 7–10 days. Plaque sizes were visualized by crystal violet staining (30–60 min) after aspiration of culture media, and cells fixation with 100% ethanol (5 min) followed by phosphate-buffered saline (PBS) wash. The plaque area was calculated (*n* = 3) using Image J which calculated an arbitrary unit value assessing the viability of the parasite with and without the protein.

*Replication assay*: The parasites were grown for two days with or without ATc (0.5 μg ml^−1^), harvested and filtered. Equal number of parasites were allowed to invade confluent HFF grown on coverslips. Following 2 h of invasion, the coverslips were washed thrice with ED1 (1% FBS containing DMEM), to remove extracellular parasites and promote synchronized replication. ATc (0.5 μg ml^−1^) was added at the start of the experiment, allowing the treatment for 24 h, alongside control parasites without ATc. These coverslips were then fixed and processed for IFA using anti-HA, anti-SAG1 antibodies wherein the parasite number per parasitophorous vacuole was analyzed.

### Electron microscopy

The *Tg*LIPIN-ikD parasites were grown for 12, 24 and 48 h in the presence and absence of ATc, in labteks (Nunk, Thermofisher). The parental ∆Ku80 strain treated with ATc was used as the wild-type control. The labteks containing parasite-infected HFF were fixed in 0.1 M cacodylate buffer with 2.5% glutaraldehyde for 2 h and kept at 4 °C until further processing. During processing, the sample were fixed again for 1 h with 1% osmium tetroxide in cacodylate buffer followed by overnight treatment in 2% uranyl acetate in distilled water. After dehydration in graded series of acetonitrile, samples were progressively impregnated in Epon812, the wells were then filled with fresh resin and allowed to polymerize 48 h at 60 °C. Ultrathin 70-nm sections were obtained with a Leica UC7 ultramicrotome and collected on copper grids. Grids were post-stained with uranyl acetate and lead citrate before their observation on a Jeol1200 EXII Transmission Electron Microscope on the Electron microscopy facility of the University of Montpellier (MEA). Chemicals and consumables were from Electron Microscopy Sciences.

### Nile red staining of lipid droplets

The *Tg*LIPIN-ikD parasites were allowed to infect and grow in confluent monolayer HFF grown on coverslips in 1% and 10% FBS, in the + /− ATc conditions for 24 h and 48 h. Similar to IFA, these coverslips were fixed using 2.5% PFA, permeabilized with 0.25% Triton X-100 and then stained with primary rat anti-HA antibody followed by detection with secondary AlexaFluor 488-conjugated goat anti-rat antibody. Thereafter, the sample coverslips were incubated for 1 h with Nile red (1:2000) in 1× PBS before proceeding to DNA staining with Hoechst. The coverslips were mounted onto a glass slide in fluorogel prior to imaging using a fluorescence microscope (Axio Imager 2_apotome; ZEISS). For visualizing Nile red-stained droplets yellow-gold fluorescence (excitation, 450-500 nm; emission, greater than 528 nm^30^) was used on the axio-imager. Quantification in + /− ATc condition was done by counting the number of lipid droplets per parasite vacuole.

### Heterologous complementation

The codon-optimized carboxy terminal sequence of *Tg*LIPIN (548-765 a.a., C-LIP) harboring the catalytic HAD domain (DVDGT), obtained from GenScript, was ligated to *NotI/MluI* digested pD0170 yeast expression vector (obtained from Carman’s lab) to yield pD0170-CLIP. This plasmid was transformed into yeast strain ∆*dpp1*∆*lpp1*∆*pah1* (*pah1*_::*URA3 dpp1*_::*TRP1/Kanr lpp1*_::*HIS3/Kanr, triple mutant*) or *Δdpp1Δlpp1Δpah1Δapp1* (*app1Δ::natMX4 pah1Δ::URA3 dpp1Δ::TRP1/Kanr lpp1Δ::HIS3*, quadruple mutant) (gifts from Dr. George Carman’s lab, Rut*g*ers Center for Lipid Research, New Jersey,), and transformants were selected on solid SD medium (YNB, 2% glucose, agar) lacking leucine^19^.

### Lipidomic analysis

#### *Saccharomyces cerevisiae*

The triple mutant strain was transformed with vector pD0170-CLIP or pDO1050 (lacking the HAD domain) and the cells were grown in YNB glucose medium at 30 °C until OD_600_ reached 1–3. Cultures were centrifuged for 5 min at 1000 × *g*, washed with PBS thrice, and normalized by cell weight.

*Total lipid analysis*: Total lipids were extracted in chloroform/methanol/water (1:3:1, v/v/v) containing FFA (free fatty acids C13:0, 10 nmol) and PC (21:0/21:0, 10 nmol) as internal standards for extraction. Next, the polar and apolar metabolites were separated by phase partitioning by adding chloroform and water to give the ratio of chloroform/methanol/water as 2:1:0.8 (v/v/v). For lipid analysis, 50 µl of the extract was directly dried and dissolved in 2:1 choloform:methanol and trimethylsulfonium hydroxide (TMSH, Machenery Nagel) for total fatty acid content. Resultant FAMEs were then analyzed by GC-MS as previously described^44^. All FAMEs were identified by comparison of retention time and mass spectra from GC-MS with authentic chemical standards. The concentration of FAMEs was quantified after initial normalization to different internal standards.

*Phospholipid, DAG/TAG analyses*: The extracted total lipid extracted (as above) was separated with 5 nmol DAG/PA(C16:0/C18:1) (Avanti Polar lipids) by one-dimensional silica gel high-performance thin-layer chromatography (HPTLC, Merck). The 1st and 2nd solvent system used were chloroform/methanol/water/ acetic acid, 25:15:2:4 (v/v/v/v) and hexane/MTBE/acetic acid, 35:15:0.5 (v/v/v), respectively. For DAG, TAG, FFA, and CE analysis, total lipid fraction was separated by 1D-HPTLC using hexane/diethyl ether/formic acid, 80:20:2 (v/v/v) as solvent system. The spots correlating to TAG, DAG, PA, and PC on the HPTLC plate were scraped off, and lipids were methanolized with 200 μl 0.5 M methanolic HCl in the presence of 1 nmol pentadecanoic acid (C15:0) as internal standard at 85 °C for 4 h. The resulting FAMEs were extracted with hexane and analyzed by GC-MS (Agilent).

#### *Toxoplasma gondii*

The parasites were grown for 24 h ad 48 h in + /− ATc conditions within a confluent monolayer of HFF in flasks (175 cm^2^). At each time point, parasites were harvested as intracellular tachyzoites (1 × 10^7^ cell equivalents per replicate) after syringe filtration with 3-μm pore size membrane. These parasites were metabolically quenched by rapid chilling in a dry ice-ethanol slurry bath and then centrifuged down at 4 °C. The parasite pellet was washed with ice-cold PBS thrice, before transferring the final pellet to a microcentrifuge tube. Then total lipids were extracted in chloroform/methanol/water (1:3:1, v/v/v) containing PC (C13:0/C13:0), 10 nmol and C21:0 (10 nmol) as internal standards for extraction. Polar and apolar metabolites were separated by phase partitioning by adding chloroform and water to give the ratio of chloroform/methanol/water as 2:1:0.8 (v/v/v). For lipid analysis, the organic phase was dried under N_2_ gas and dissolved in 1-butanol to obtain 1 µl butanol/10^7^ parasites.

*Total lipid analysis*: The extracted total lipid sample was then added with 1 nmol pentadecanoic acid (C15:0) as an internal standard as stated before using TMSH for total fatty acid content. Resultant FAMEs were analyzed by GC-MS as previously described^44^. All FAMEs were identified by comparison of retention time and mass spectra from GC-MS with authentic chemical standards. The concentration of FAMEs was quantified after initial normalization to different internal standards and finally to parasite number.

*Free fatty acid and cholesterol analysis*: Total lipid samples were dried and derivatized with BSTFA + TMCS, 99:1 (Sigma) to generate trimethylsilyl (TMS-) fatty acids and TMS-cholesterol. These TMS derivatives were analyzed by GCMS as described above.

*Phospholipid and neutral lipid analysis*: For phospholipid analysis, the extracted **t**otal lipid extracted (as above) was separated with 1 nmol PA(C17:0/C17:0) (Avanti Polar lipids) by two-dimensional silica gel high-performance thin-layer chromatography (HPTLC, Merck). The solvent system used for the 1st and 2nd dimension was chloroform/methanol/28% ammonium hydroxide,12:7:1.6 (v/v) and chloroform/acetone/methanol/acetic acid/water, 10:4:2:2.6:1 (v/v/v/v/v), respectively. For DAG, TAG, Free fatty acids (FFA) and cholesteryl ester (CE) analysis, total lipid fraction was separated by 1D-HPTLC using hexane/diethyl ether/formic acid, 80:20:2 (v/v/v) as solvent system. Then each lipid spot on the HPTLC plate was scraped off, and lipids were methanolized with 200 μl 0.5 M methanolic HCl in the presence of 1 nmol pentadecanoic acid (C15:0) as internal standard at 85 °C for 3 h. The resulting FAMEs were extracted with hexane and analyzed by GC-MS (Agilent).

### Stable isotope metabolic labeling experiment

#### Tracking FASII origin fatty acids (monitoring de novo FA synthesis by the parasite apicoplast FASII)

The *Tg*LIPIN parasites were infected to a confluent monolayer of HFF in glucose-free-DMEM (1% FBS) supplemented with U-^13^C-glucose or U-^12^C-glucose at a final concentration of 800 µM, with or without ATc (0.5 μg ml^−1^). The parasites were harvested up to 48 h post depletion of *Tg*LIPIN and metabolically quenched in a dry ice and ethanol slurry in a tube until the sample reached 4 °C. Lipids were extracted, derivatized using TMSH (Macherey-Nagel) and analyzed by GC-MS as described above. ^13^C incorporation to each fatty acid was calculated as the percent of the metabolite pool containing one or more ^13^C atoms after correction for natural abundance and the amount of ^13^C-carbon source in the culture medium. The degree of the incorporation of ^13^C into fatty acids (% carbon incorporation) was determined by the mass isotopomer distribution (MID) of each FAMEs. MID was obtained from the shift in isotopic mass dependent on the amount of ^12^C carbons compared to the integration of ^13^C carbon atoms. The total abundance of ^13^C-labeled fatty acids was obtained by calculating the concentration of all isotopomers of ^13^C-labeled FAMEs and finally normalizing to authentic internal standards and parasite number.

#### Tracking host-derived fatty acids (monitoring parasite scavenging capacities)

The HFF cells were grown (1 × 10^8^ cell equivalents per replicate) to confluency in the presence of stable isotope U-^13^C-glucose at a final concentration of 800 µM added to a glucose-free DMEM. These ^13^C-pre-labeled HFF were then infected with *Tg*LIPIN-ikD parasites in the presence of normal-glucose containing DMEM under + /− ATc (0.5 μg/ml). The host HFF and parasites were metabolically quenched separately, and their lipid content was quantified by GC-MS as described above. As described previously, the degree of the incorporation of ^13^C into fatty acids (%carbon incorporation) is determined by the mass isotopomer distribution (MID) of each FAMEs. The total abundance of ^13^C-labeled fatty acids was analyzed initially for HFF to check labeling of the metabolites (described previously). Later, the same was calculated for parasites to confirm direct uptake of ^13^C-labeled fatty acids from the host.

#### Tracking uptake of deuterated fatty acid from medium (monitoring extracellular host environment)

Deuterated palmitic acid (^31^d-C16:0) was dissolved in 10 mM in fatty acid-free bovine serum albumin/PBS solution by sonication in a water bath for 30 min followed by incubation at 55 °C for 30 min. Freshly egressed *Tg*LIPIN parasites were allowed to invade a confluent monolayer of HFF for at least 2 h under conditions of +/− ATc. Following the invasion, the uninvaded parasites were washed off with DMEM and further allowed to grow in the normal culture medium-DMEM (1% FBS) containing ^31^d-C16:0 at a final concentration of 0.1 mM in +/− ATc until 24 and 48 h of growth. The parasites were harvested by metabolic quenching as described previously. Lipids were extracted, derivatized using TMSH as well as TMS, and further analyzed by GC-MS. All raw data were quantitatively and qualitatively analyzed using Agilent MassHunter Software: MS B.07.00.

### Statistics and reproducibility

The graphical data for this study was generated using GraphPad Prism software. Three biological replicates were used per experiment (*n* = 3, unless stated otherwise). The error bars are representative of the standard error of mean (SEM) for each study. Statistical significance was determined for each experiment by unpaired *t* tests using GraphPad Prism. The range of statistical significance was signified as per the *P* value, where 0.01–0.05 = *, 0.01–0.001 = **, <0.001 = ***, and <0.0001 = ****.

### Reporting summary

Further information on research design is available in the Nature Research Reporting Summary linked to this article.

## Supplementary information

Supplementary Information

Reporting Summary

## Data Availability

Authors can confirm that all relevant data are included in the paper and/or its Supplementary/Source data files. Protein and gene sequences were acquired from EuPathDB web sources ToxoDB (http://toxodb.org/toxo/). Source data are provided with this paper.

## Source data

Source Data

## Code availability

No codes were used to analyze the data in this study.
