# Supplementary material for: Toxoplasma LIPIN is essential in channeling host lipid fluxes through membrane biogenesis and lipid storage
Source: Nat Commun. 2021 May 17;12:2813. doi: 10.1038/s41467-021-22956-w (PMC8129101; doi:10.1038/s41467-021-22956-w)
Supplement: Supplementary file 1 — Supplementary Information [file 41467_2021_22956_MOESM1_ESM.pdf]

# Supplementary Information

## *Toxoplasma* LIPIN is essential in channeling host lipid fluxes through membrane biogenesis and lipid storage

Sheena Dass*[1], Serena Shunmugam*[1], Laurence Berry[2], Christophe-Sebastien Arnold[1], Nicholas J. Katris[1], Samuel Duley[1], Fabien Pierrel[3], Marie-France Cesbron-Delauw[1], Yoshiki Yamaryo-Botté[1]**, Cyrille Y. Botté[1]**

1 Apicolipid Team, Institute for Advanced Biosciences, CNRS UMR5309, Université Grenoble Alpes, INSERM U1209, Grenoble, France,
2 Laboratory of Pathogen Host Interactions, UMR 5235, Université de Montpellier, France.
3 Université Grenoble Alpes, CNRS, Grenoble INP, TIMC-IMAG, 38000 Grenoble, France
* These authors contributed equally
** These authors jointly supervised this work.
To whom correspondence should be sent, cyrille.botte@univ-grenoble-alpes.fr/cyrille.botte@gmail.com, yoshiki.botte-yamaryo@univ-grenoble-alpes.fr

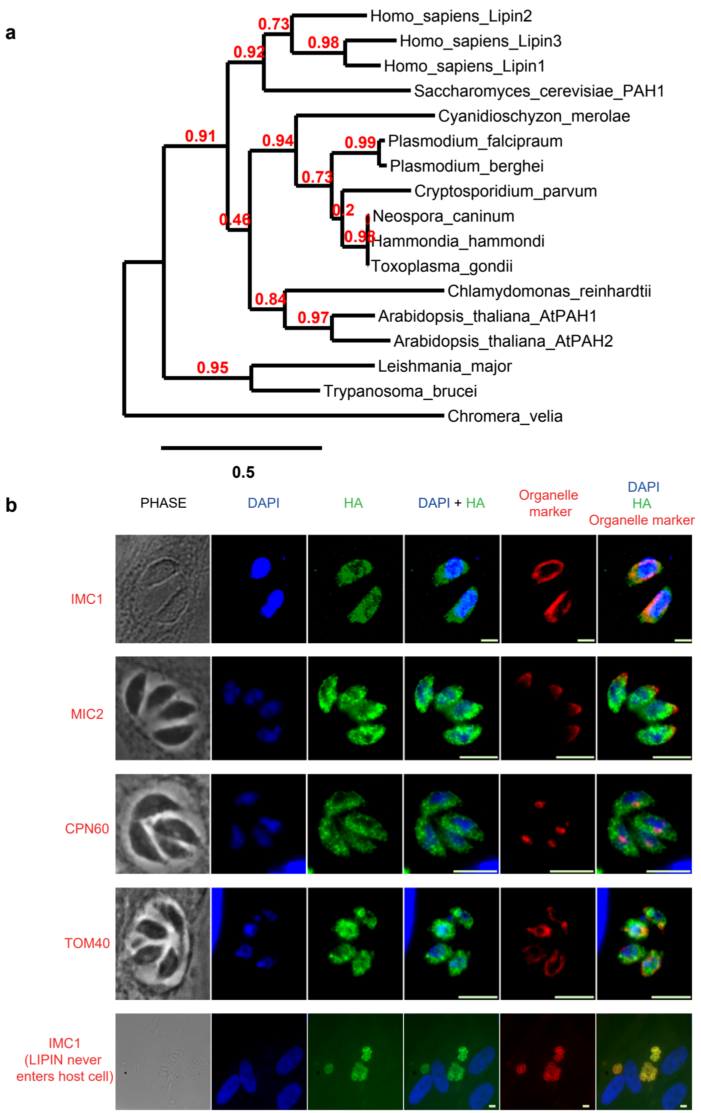


**Supplementary fig. 1 a)** Phylogenetic tree of the eukaryotic lipin homologs of *Tg*LIPIN. Entire protein sequence was used to create the tree using web server Phlogeny.fr. **b)** IFA analyses of *Tg*LIPIN with anti-HA, DAPI and IMC1/MIC2/CPN60/TOM40 antibodies, shows disperse cytoplasmic and perinuclear localization of the enzyme, which also does not extend to the host cell. Scale bar: 2 µm, n=3.

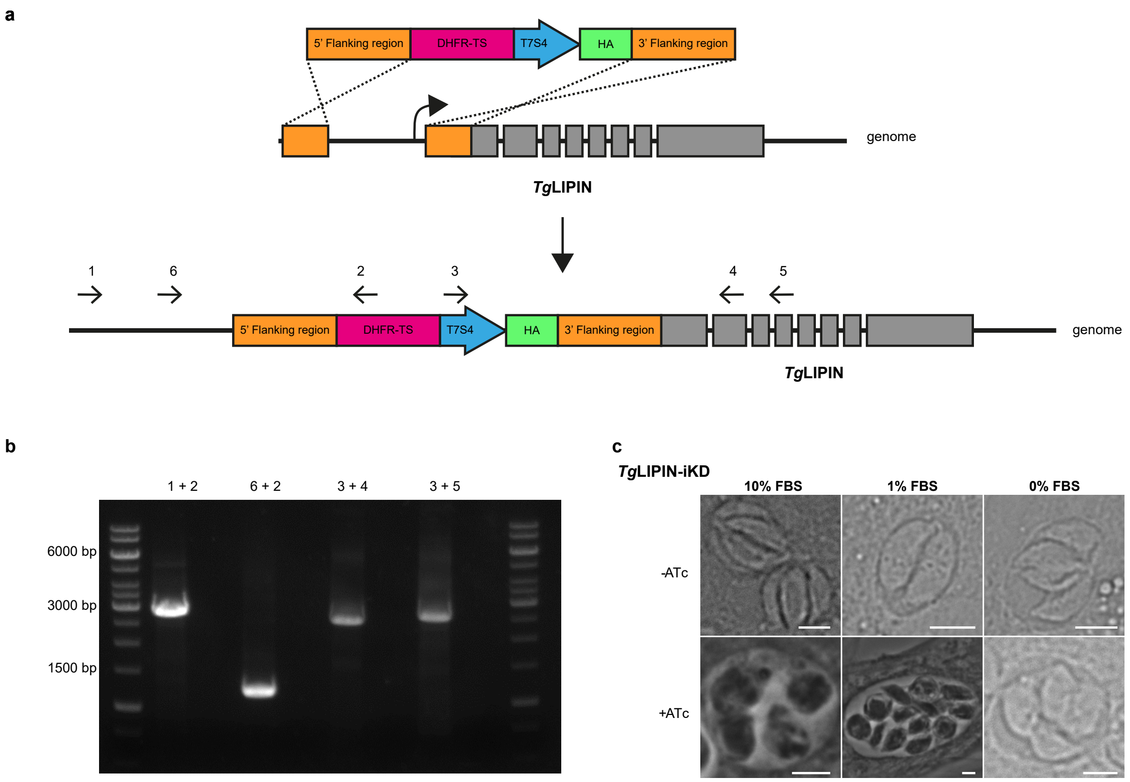


**Supplementary fig. 2 a)** Schematic of molecular strategy for generation of *Tg*LIPIN-ikD tagged with 3X HA at its N-terminus. **b)** Confirmation of correct promoter replacement in the *Tg*LIPIN-ikD line. Primers combinations during the screening PCR yielded products of expected amplicon size validating the mutant. The template genomic DNA was extracted from *Tg*LIPIN-ikD line Primers 1 + 2/6+2 generated 2893/1153 bp amplicon with *Tg*LIPIN-ikD gDNA suggesting correct incorporation of tetracycline inducible elements along with DHFR cassette at the genome locus of *Tg*LIPIN. Expectedly, primers combinations specific to Tet-regulatable elements and the gene locus (3+4/3+5) generated an amplicon 2590/2685 bp in size using the genomic DNA of *Tg*LIPIN as the template. **c)** Brightfield image of the phenotypic effects of *Tg*LIPIN depletion after 48h (-/+ATc) at 10%, 1%, and 0% FBS. Scale bar: 5.0 µm, n=3.

**Supplementary Table 1: Primer list used in this study**

| Primer name | Sequence |
|---|---|
| F1 | 5’- TACTTCCAATCCAATTTAATGCACGGCAGATTTCTCTTACTGG -3’ |
| R1 | 5′- TGGATCCGGCGCGCCATGCATCCTGGACCAGAGAGGAAAAGAG -3’ |
| F2 | 5′- TGTTCCAGATTATGCCTTACCCGGGATGTGGGGGAAGATTGTCTCGAGC -3’ |
| R2 | 5′- GCACTGACTGGCATGAATGGCCAGGCGCTGCCTTCTTTCCATTC- 3’ |
| Screen Primer 1 | 5’- CGATGACCTGTGTCGACCTGT -3’ |
| Screen Primer 2 | 5′- TCTTCTTTGAGGGAAGAGGAAACG -3’ |
| Screen Primer 3 | 5’- GGTACCGAGCTCGACTTTCAC -3’ |
| Screen Primer 4 | 5’- CAGCTGATCGGAGGTTGGTCT -3’ |
| Screen Primer 5 | 5’- ACTCTCAACCACACCACACATCC -3’ |
| Screen Primer 6 | 5’- CTCCACCGTTTCCGGTTCCGT -3’ |

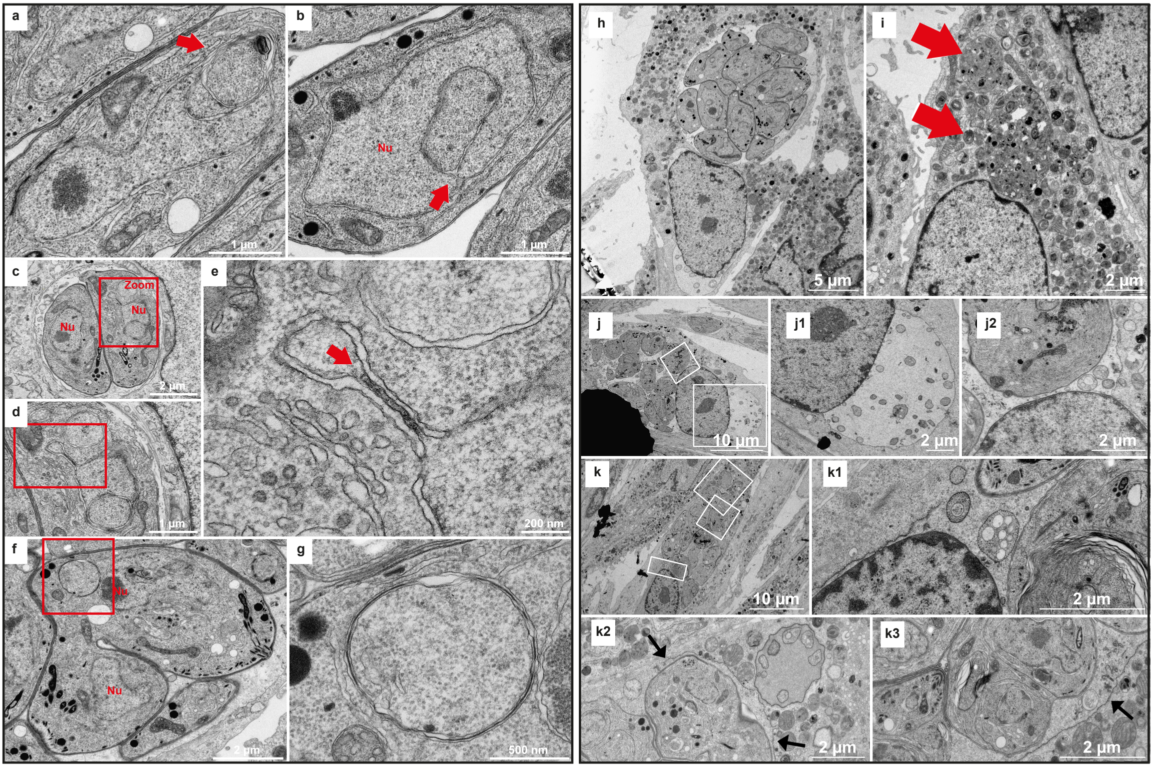


**Supplementary fig. 3** Details of nuclear membrane alterations observed in *Tg*LIPIN KD parasites 24 hours after induction. **a)** The nuclear membrane elongates and makes whorls in the cytoplasm. **b)** Local fusion of the outer nuclear membrane between different lobes was observed (red arrow). **c-e)** Nuclear membrane elongations make hairpins and wraps **c)** and higher magnifications in **d)** and **e)**, showing large zones where the inner nuclear membrane from different parts of the nucleus are in close apposition. **e)** Higher magnification shows that the nucleoplasmic leaflet of the inner membrane seems to be detached and shows partial fusion with the leaflet of the other side of the hairpin (red arrow). **f-g)** Nuclear membrane elongations make zippered structures formed by a double layer of nuclear membrane tightly apposed. Nu: Nucleus; **h-k)** The host cell did not show signs of apoptosis or necrosis, however structures previously described as "gMVB" and formed by the swelling of the nuclear envelope or the ER of the host cell (Amiar *et al.* 2020) were frequently observed in the *Tg*LIPIN KD parasites (**i,** red arrow, **j1**, **k2**). **j2)** In presence of 10% FBS, fusion of the parasitophorous vacuole with the nuclear swollen nuclear envelope of the host cell could be observed (red arrow). **k1-3)** Occasionally, this led to the rupture of the outer membrane of the host cell nuclear envelope. Fragments of the disrupted nuclear envelope membrane with ribosome are pointed with black arrows at higher magnification in **k1**, **k2**, and **k3**. At 48h post induction, large membrane-bound cytoplasmic inclusions were frequently observed (**b**, red arrows) in the cytoplasm of the HFFs. Even though the content of theses vacuoles cannot be clearly identified, they likely result from degradation of parasite containing vacuoles, following parasite necrosis-like process, and/or fusion of the vacuole with lysosomes.

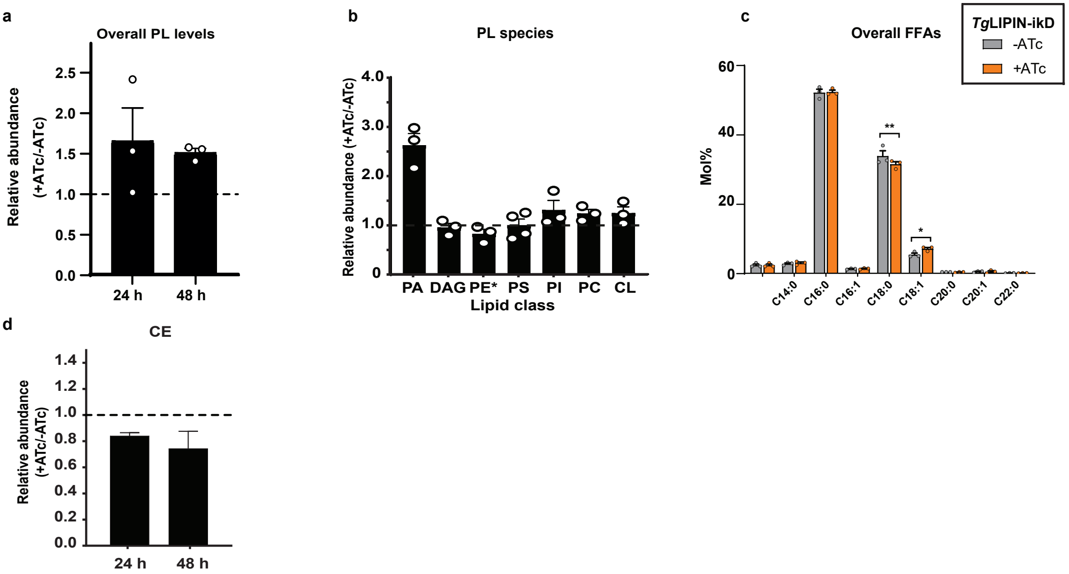


**Supplementary fig. 4 a)** Overall relative abundance of phospholipid levels at 24 h and 48 h, showing no changes without/with *Tg*LIPIN (+ATc/-ATc) **b)** Relative abundance of major phospholipid species, PA, PS, PC and CL, showed a significant increase in the *Tg*LIPIN-ikD mutant (+ATc, orange) in comparison to the control (-ATc, grey). The biochemical product of *Tg*LIPIN, DAG was reduced in the *Tg*LIPIN-ikD mutant (+ATc) whereas, phospholipids PE, PI remained unchanged. **c)** Composition of the molecular species within free fatty acid (FFA) content of *Tg*LIPIN-ikD (+ATc/-ATc), showing a significant increase of oleate (C18:1) upon *Tg*LIPIN depletion. **d)** The relative abundance (+ATc/-ATc) of cholesteryl esters (CE) remained unaffected upon depletion of *Tg*LIPIN. Experiments were conducted in triplicates, unpaired t-test p values are indicated wherein 0.01-0.05=*, 0.01-0.001=**, <0.001=*** and <0.0001=****.

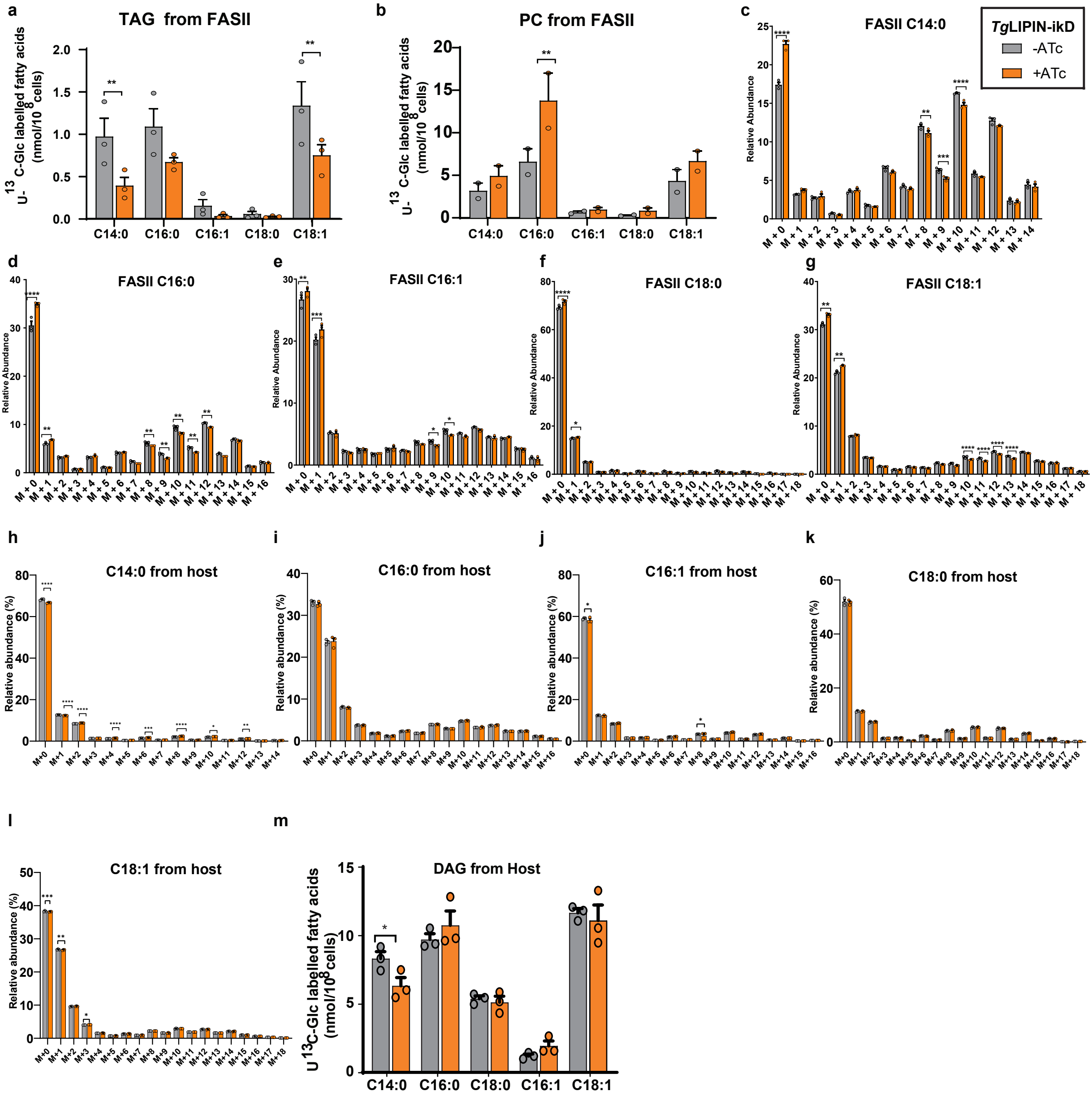


**Supplementary fig. 5 a)** The graph represents U13-C-labelled of FASII-derived fatty acid species within TAG levels of *Tg*LIPIN-ikD, showing a decrease in C14:0 and C18:1. **b)** Graphical representation of the decrease in FASII-derived PC, specifically oleic acid (18:1). Mass isotopologue distribution of C14:0, C16:0, C16:1, C18:0 and C18:1 fatty acid species **c-g)** *de novo* synthesized and **h-l)** scavenged directly from the host. **m)** U13-C-labelled of host-derived fatty acid species within DAG levels of *Tg*LIPIN-ikD. Experiments were conducted in triplicates, unpaired t-test *p* values where 0.01-0.05=*, 0.01-0.001=**, <0.001=*** and <0.0001=****.
