# Supplementary material for: Toxoplasma LIPIN is essential in channeling host lipid fluxes through membrane biogenesis and lipid storage
Source: Nat Commun. 2021 May 17;12:2813. doi: 10.1038/s41467-021-22956-w (PMC8129101; doi:10.1038/s41467-021-22956-w)
Supplement: Supplementary file 2 — Reporting Summary [file 41467_2021_22956_MOESM2_ESM.pdf]

nature research

Corresponding author(s): Cyrille Y. BOTTE, Yoshiki YAMARYO-BOTTE

Last updated by author(s): Mar 12, 2021

Reporting Summary

Nature Research wishes to improve the reproducibility of the work that we publish. This form provides structure for consistency and transparency in reporting. For further information on Nature Research policies, see our Editorial Policies and the Editorial Policy Checklist.

Statistics

For all statistical analyses, confirm that the following items are present in the figure legend, table legend, main text, or Methods section.

n/a	Confirmed	
☐	☒	The exact sample size (*n*) for each experimental group/condition, given as a discrete number and unit of measurement
☐	☒	A statement on whether measurements were taken from distinct samples or whether the same sample was measured repeatedly
☐	☒	The statistical test(s) used AND whether they are one- or two-sided<br>*Only common tests should be described solely by name; describe more complex techniques in the Methods section.*
☒	☐	A description of all covariates tested
☒	☐	A description of any assumptions or corrections, such as tests of normality and adjustment for multiple comparisons
☐	☒	A full description of the statistical parameters including central tendency (e.g. means) or other basic estimates (e.g. regression coefficient) AND variation (e.g. standard deviation) or associated estimates of uncertainty (e.g. confidence intervals)
☐	☒	For null hypothesis testing, the test statistic (e.g. *F*, *t*, *r*) with confidence intervals, effect sizes, degrees of freedom and *P* value noted<br>*Give P values as exact values whenever suitable.*
☒	☐	For Bayesian analysis, information on the choice of priors and Markov chain Monte Carlo settings
☒	☐	For hierarchical and complex designs, identification of the appropriate level for tests and full reporting of outcomes
☒	☐	Estimates of effect sizes (e.g. Cohen's *d*, Pearson's *r*), indicating how they were calculated

Our web collection on statistics for biologists contains articles on many of the points above.

Software and code

Policy information about availability of computer code

Data collection	Agilent MassHunter Software: MS quantitative analyses and MS qualitative analyses B.07.00, PhyML 3.1/3.0 aLRT
Data analysis	Microsoft Office Exceland GraphPad Prism 8.0, ImageJ (NIH)

For manuscripts utilizing custom algorithms or software that are central to the research but not yet described in published literature, software must be made available to editors and reviewers. We strongly encourage code deposition in a community repository (e.g. GitHub). See the Nature Research guidelines for submitting code & software for further information.

Data

Policy information about availability of data

All manuscripts must include a data availability statement. This statement should provide the following information, where applicable:

- Accession codes, unique identifiers, or web links for publicly available datasets
- A list of figures that have associated raw data
- A description of any restrictions on data availability

The authors declare that [the/all other] data supporting the findings of this study are available within the paper [and its supplementary information files].

Field-specific reporting

Life sciences study design

All studies must disclose on these points even when the disclosure is negative.

Sample size	Since the study involves comparison between wild type parasites and knock out mutant parasites, a minimum of 3 biological replicates were chosen for each separate experiment. Beyond this, several technical replicates were generated to ensure data reproducibility. Student-t tests were used to determine statistical significance. The sample size chosen was experimentally feasible.
Data exclusions	No data were not excluded
Replication	All experiments where preformed in triplicate, SEM are indicated in the graphs, statistical significance were determined by two-sided student t.test
Randomization	Randomization does not apply to our study as we have been analyzing control (i.e. parental/wt) vs knock-out mutants.
Blinding	Blinding was not relevant since it was not practically feasible in terms of experimentation execution, we have been analysing all data in comparison to the wild-type hence it cannot be blined.

Reporting for specific materials, systems and methods

We require information from authors about some types of materials, experimental systems and methods used in many studies. Here, indicate whether each material, system or method listed is relevant to your study. If you are not sure if a list item applies to your research, read the appropriate section before selecting a response.

Materials & experimental systems

n/a	Involved in the study	
☐	☒	Antibodies
☐	☒	Eukaryotic cell lines
☒	☐	Palaeontology and archaeology
☒	☐	Animals and other organisms
☒	☐	Human research participants
☒	☐	Clinical data
☒	☐	Dual use research of concern

Methods

n/a	Involved in the study	
☒	☐	ChIP-seq
☒	☐	Flow cytometry
☒	☐	MRI-based neuroimaging

Antibodies

Antibodies used

anti-HA (Rat clone 3F10, Roche) #LOT 27573500
anti-TOM40 (Rabbit, kind gift from Dr. Giel G Van Dooren, ANU Australia who generated it)
anti-IMC1 (Mouse, kind gift from Prof Dominique Soldati-Favre, University of Geneva)
AlexaFluor 488- and 546-conjugated anti-rat (Life Technologies #LOT 1887148) and anti-rabbit antibodies (Life Technologies #LOT 1246464)
anti-MIC2 (Rabbit, kind gift provided by Prof Dominique Soldati, Universite Geneve, and made by Prof David Sibley, Washington University)
Anti-SAG1 (mouse, AbCAM, ref :8313)
Goat anti- Rabbit and anti mouse Horse Radish Peroxydases conjugated secondary antibodies were purchased from Thermo Scientific (ref # 31430 for the anti mouse, and ref#31460 for the anti-Rabbit)

Validation

anti-HA (High affinity from Sigma, validation on: https://www.sigmaaldrich.com/catalog/product/roche/roahaha?lang=en&region=FR&gclid=Cj0KCQiAv6yCBhCLARIsABqJTjaEI4yu0px3VOZ1wU-MExas4WZxXVcwTA3UACBT4HsDXVM5DoCpimAaAgYCEALw_wcB) : This was also validated on T. gondii in the following reference Jennifer Jung et. al Molecular and cellular biology, 35(14), 2015
anti-TOM40: Generated and validated in the following reference: Giel G Van Dooren et al Journal of Biological Chemistry doi: 10.1074/jbc.M116.725069, 2016
anti-IMC1: validated in (Amiar and Katris et al Cell Reports 2020 (Mar 17;30(11):3778-3792.e9. doi: 10.1016/j.celrep.2020.02.072.)
Alexa Fluor 488- : validated by supplier (https://www.thermofisher.com/antibody/product/Goat-anti-Mouse-IgG-IgM-H-L-Secondary-Antibody-Polyclonal/A-10680) and 546-conjugated (validated by supplier: https://www.thermofisher.com/antibody/product/Goat-anti-Rabbit-IgG-H-L-Cross-Adsorbed-Secondary-Antibody-Polyclonal/A-11010) anti-rat and anti-rabbit antibodies: Both were also validated in Amiar et al 2016 Plos Pathogens DOI:10.1371/journal.ppat.1005765, Amiar and Katris et al Cell Reports 2020 (accepted)
Anti-MIC2 was validated in the following reference: Amiar and Katris et al. Cell Reports Mar 17;30(11):3778-3792.e9. doi: 10.1016/j.celrep.2020.02.072.
Anti-SAG1 (mouse) validated by supplier : https://www.abcam.com/toxoplasma-gondii-antibody-tp3-ab8313.html
Goat anti-Mouse and anti- Rabbit HRP conjugated are validated by the supplier (https://www.abcam.com/toxoplasma-gondii-antibody-tp3-ab8313.html ; https://www.thermofisher.com/antibody/product/Goat-anti-Mouse-IgG-H-L-Secondary-Antibody-Polyclonal/31430)

Eukaryotic cell lines

Policy information about cell lines

Cell line source(s)	T. gondii RH TATi1-DeltaKu80 (Sheiner et al., 2011) obtained from Dr Giel Van Dooren (University of Canberra) and Human foreskin fibroblast cells (ATCC-CCL-171) obtained from Dr Corinne Mercier (Universite Grenoble Alpes)
Authentication	All parasite mutants (tagged or otherwise) were confirmed by immunoblot and immunofluorescence,
Mycoplasma contamination	Regular mycoplasma PCR-check: always negative
Commonly misidentified lines (See ICLAC register)	No commonly mistaken lines were used in this study
